# Synergistic Effect of Nitrogen Doping and MWCNT Intercalation for the Graphene Hybrid Support for Pt Nanoparticles with Exemplary Oxygen Reduction Reaction Performance

**DOI:** 10.3390/ma11040642

**Published:** 2018-04-22

**Authors:** Kang Fu, Yang Wang, Ying Qian, Linchang Mao, Junhong Jin, Shenglin Yang, Guang Li

**Affiliations:** State Key Laboratory for Modification of Chemical Fibers and Polymer Materials, College of Materials Science and Engineering, Donghua University, Shanghai 201620, China; fuk_1992@126.com (K.F.); DHUWangY@163.com (Y.W.); viskyying@163.com (Y.Q.); 2140278@mail.dhu.edu.cn (L.M.); jhkin@dhu.edu.cn (J.J.); slyang@dhu.edu.cn (S.Y.)

**Keywords:** oxygen reduction reaction, graphene–multi-walled carbon nanotubes, N doping, proton exchange membrane fuel cells

## Abstract

The potential of graphene–multi-walled-carbon nanotube (G-M) hybrids prepared by the one-pot modified Hummers method followed by thermal annealing has been demonstrated by employing one as an electrocatalyst support for oxygen reduction reaction (ORR). N doping effectively modified the electronic structure of the G-M hybrid support, which was beneficial for the uniform distribution of Pt nanoparticles, and ORR activities were further improved. The newly prepared Pt/N-G-M catalyst demonstrated higher electrochemical activity than Pt/G-M and Pt/G catalysts. Even compared with commercial 20 wt % Pt/C (JM20), Pt/N-G-M delivered a better half-wave potential and mass activity. In terms of the durability test, Pt/N-G-M maintained 72.7% of its initial electrochemical active surface area (ECSA) after 2000 repeated potential cycles between 0 and 1.2 V in acidic media in relation to the 44.4% retention for JM20. Moreover, the half-wave potential for Pt/N-G-M showed only a minimal change, significantly superior to the 139 mV of loss for JM20. It is expected that Pt/N-G-M can be the potential candidate as a highly efficient and durable catalyst if utilized in proton exchange membrane fuel cells (PEMFCs).

## 1. Introduction

In the context of increasing worldwide energy demand and environmental pollution, fuel cells are an eye-catching arena owing to their exciting performance for power generation with low emission [1,2]. Among the multitude of fuel cell technologies available, proton exchange membrane fuel cells (PEMFCs) have recently received extensive attention owing to their high efficiency and power density [3], appealing for automotive and portable electronic applications [2]. Nevertheless, challenging issues including the high cost, insufficient oxygen reduction reaction (ORR) activity, and durability of the cathode catalysts are critical obstacles that hinder their practical commercial viability [4,5]. Precious metals such as Pt or Pt-based alloys are routinely utilized as cathode catalysts due to their high catalytic performance towards ORR [6]. To fully use these precious metal catalysts in fuel cells cost-effectively, they have been supported on nanostructrured carbonaceous materials [7]. State-of-the-art commercial catalysts in PEMFCs are still based on platinum nanoparticles dispersed on carbon black (CB). Problems plaguing such Pt/C catalysts, including the degradation of carbon [8] and the agglomeration and dissolution of Pt nanoparticles [9,10] lead to the deactivation of catalysts. Recently, carbon nanotubes [11,12], carbon nanofibers [13,14,15], and mesoporous carbon [16,17,18] have been systematically exploited as the substitutes of CB in ORR catalysts. As a new star member of carbon materials, graphene (G) has attracted worldwide attention since its rise [19]. The high theoretical specific surface area, ultrahigh electrical conductivity, fast available electron transfer, and high durability to electrochemical corrosion of G open new avenues for electrocatalyst supports [20]. Thus, a substantial research effort has been put into G-based metal catalysts towards ORR, and great strides have been reported [21,22].

Despite the known advantages, graphene materials are susceptible to aggregation and/or restacking to form graphite because of the strong π–π interactions and interlayer Van der Waals forces, which limit the optimization of the electrocatalytic performance of graphene [23]. The agglomerate structure results in a significant decrease in supporting area and mass transfer, inevitably retarding the utilization of catalysts [24]. In spite of the tremendous research on the promising approach of incorporating spacers into graphene nanosheets [25,26], they often involve in multistep procedures and the interactions between them are not that satisfactory. So far, the effective and facile methods to prevent the restacking of graphene sheets are still underway.

In addition, it is well-recognized that introducing heteroatom N into the graphitic carbon structure contributes to tailoring the underlying catalyst–support interactions to boost the electrocatalytic activity and stability [27]. There have been extensive reports on nitrogen-doped graphene-supported catalysts, the results of which all demonstrate improved ORR activity and durability, or higher membrane electrode assembly (MEA) performance [28,29,30,31,32]. For example, He et al. [29] obtained nitrogen-doped reduced graphene oxide (NRGO) with a nitrogen content of 5.06%, revealing a higher ORR performance. Vinayan et al. [30] proved that the power density of MEA for Pt_3_Co/N-HEG catalyst increases by four times compared with commercial Pt/C catalyst. 

Inspired by the above, herein, a novel multi-walled carbon nanotube (MWCNT) and graphene (G-M) hybrid support was constructed through the one-pot modified Hummers method, which was followed by carbonization [33]. The MWCNT increased the basal spacing between graphene sheets, providing a high specific surface area. Heteroatom N was then doped into the hybrid support through a facile approach using dicyandiamide (DCDA) as the N-containing precursor. Pt nanoparticles were deposited on different supports via a facile ethylene glycol reduction technique, and the electrocatalytic performance was investigated and is discussed here in detail. 

## 2. Experimental

### 2.1. Material Synthesis 

**Figure 1:**
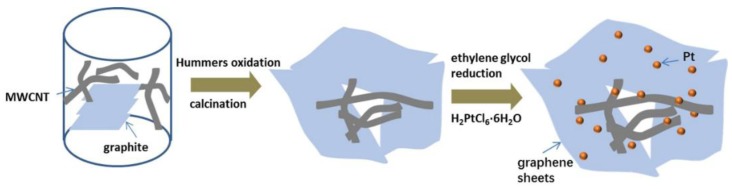
The procedures showing the preparation of Pt/G-M.

As described previously, a modified Hummers method was used to synthesize graphene oxide (GO) from the 325 mesh graphite (Aladdin, Shanghai, China) [34]. The typical procedure of preparing a G-M hybrid and depositing Pt nanoparticles is depicted in Figure 1. In a typical synthesis, flaky graphite and commercially available MWCNTs (Chengdu Organic Chemicals Co. Ltd., Chengdu, China; with a diameter of 10–20 nm and a Brunauer–Emmett–Teller (BET) surface area of ~200 m^2^·g^−1^) were mixed together in a 4:1 mass ratio through a one-pot modified Hummers method followed by carbonization at 1000 °C for 1 h in an N_2_ atmosphere [33]. For comparison, G was also thermally reduced under identical conditions as a benchmark and N-modification of the G-M (N-G-M) was performed by grinding G-M and dicyandiamide (DCDA) (mass ratio *m/m* = 1:12) and subsequent calcination at 900 °C for 2 h.

Pt nanoparticles were supported on the as-obtained support materials by an ethylene glycol reduction method [35]. Briefly, 40 mg of carbon support materials were added into the ethylene glycol and water (*v/v* = 3:1) solution, and a certain amount of H_2_PtCl_6_·6H_2_O was then added to ensure a Pt content of 20 wt %. The slurry was refluxed at 140 °C and maintained for 4 h under magnetic stirring with the protection of N_2_ atmosphere. After centrifuging with water and acetone, drying in a vacuum oven overnight, the obtained samples were collected. They were named as Pt/M, Pt/G, Pt/G-M, and Pt/N-G-M, corresponding to the MWCNT, G, G-M, and N-G-M supports, respectively. In the meantime, commercial 20 wt % Pt/C noted as JM20 purchased from the Johnson Matthey Company (Royston, UK) was employed as the control sample.

### 2.2. Structural Characterization

The morphologies of carbon supports were observed with a field-emitting scanning electron microscope (FESEM, Hitachi SU-8010, Hitachi, Tokyo, Japan). Transmission electron microscopy (TEM, JEOL-2100F, JEOL Ltd., Tokyo, Japan) was carried out to observe the morphologies of the supports and for Pt nanoparticle size and distribution. Nitrogen adsorption–desorption isotherm was collected on an ASAP2020 volumetric adsorption analyzer (Micromeritics, Norcross, GA, USA) at –196 °C. Raman spectra of the supports were obtained on a Via-Reflex microscopic confocal Raman spectrometer (Renishaw, Wotton-under-Edge, UK). Wide powder X-ray diffraction (XRD, Bruker, Billerica, MA, USA) patterns of all catalysts were determined on a Bruker-D8-AXS diffractometer with Cu Kα radiation. X-ray photoelectron spectrometry (XPS, Rbd Instruments, Bend, OR, USA) studies were measured on a PHI-5400 spectrometer equipped with Mg Ka (hν = 1253.6 eV) to evaluate the chemical states of the surface elements in the catalysts. The Pt content was tested via inductively coupled plasma-atomic emission spectroscopy (ICP-AES, Prodigy, Teledyne Leeman Labs, Hudson, NH, USA). The Pt loadings for the Pt/M, Pt/G, Pt/G-M, and Pt/N-G-M catalysts were about 19.3, 20.8, 21.2, and 20.4 wt %, respectively.

### 2.3. Electrochemical Measurements

The electrochemical performance of the as-prepared catalysts was examined by the cyclic voltammetry (CV) and linear sweep voltammogram (LSV) measurements on a CHI760E Electrochemical Analyzer (CH Instruments Inc., Shanghai, China) coupled with a Pine Modulated Speed Rotator. A traditional three-electrode system was used, in which a graphite rod and saturated Hg/HgO electrode were the counter electrode and the reference electrode, respectively, and a glassy carbon rotating disk electrode (RDE, Pine Research Instrumentation, Durham, NC, USA) was the working electrode. For convenience, the potentials refer to the reversible hydrogen electrode (RHE) in this study. The uniform catalyst ink (JM20, Pt/M, Pt/G, Pt/G-M, and Pt/N-G-M) was fabricated by adding 4 mg of the catalyst into 2 mL of the methanol/Nafion solution (50:1 in weight) followed by ultrasonication for 1 h. Then, 20 µL of ink was deposited onto the RDE to get a Pt loading of 32.39 μg_Pt_·cm^−2^. CV measurements were performed in 0.1 M HClO_4_ at a potential scan rate of 50 mV·s^−1^ between 0 and 1.2 V, and the HClO_4_ solution was before saturated with high pure N_2_. LSV measurements for the ORR polarization were tested by the RDE technique in O_2_-saturated 0.1 M HClO_4_ electrolyte with an electrode rotating speed of 1600 rpm at 5 mV·s^−1^. For the test of methanol tolerance, 0.1 M CH_3_OH was added into the 0.1 M HClO_4_ solution. To evaluate the long-term stabilization of the catalysts, accelerated durability tests (ADTs) were carried out in an N_2_-saturated 0.1 M HClO_4_ solution by cycling in a potential range from 0 to 1.2 V versus RHE at 100 mV·s^−1^ for 2000 cycles. All the tests were carried out at room temperature. 

## 3. Results and Discussion

**Figure 2:**
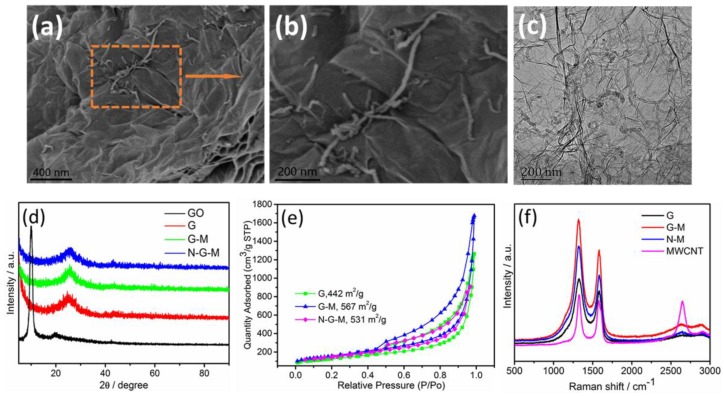
FESEM images for G-M (**a**,**b**); TEM image of G-M (**c**); XRD patterns of GO, G, G-M, and N-G-M (**d**); N_2_ adsorption–desorption isotherms of G, G-M, and N-G-M supports (**e**); Raman spectra of MWCNT, G, G-M, and N-G-M supports (**f**).

As illustrated in Figure 1, the G-M hybrid support was synthesized via a one-pot modified Hummers method and subsequent calcination. N-modification was conducted by carbonization of the mixture of G-M and DCDA. Successful preparation of G-M and N-G-M hybrid supports was confirmed by SEM, TEM, and XRD. In comparison to the simple mixing of GO and spacer [36,37] or with the help of dispersing agent [38], we creatively involved MWCNTs and graphite in the Hummers method. In the synthesis of hybrid supports, the functionalization of MWCNTs by strong oxidants to form various hydrophilic moieties accompanied by partial unzipping and peeling (Figure S1) enables its incorporation with GO. Even after carbonization, the three-dimensional (3D) structure remained, with MWCNT still inserted into the G sheets. MWCNT is well-distributed between the G layers without obvious aggregation. Figure 2a–c clearly displays the 3D interconnected structure of G-M composed of wrinkled G and 1D MWCNTs, which may be a promising support for ORR catalysts [39,40]. Figure 2d shows the XRD patterns of G, G-M, and N-G-M supports compared with GO. The diffraction peak at 26.0° for G, G-M, and N-G-M is indexed to the C (002) reflection of carbon and the disappearance of peak at 10° suggests the reduction of GO. The same peak for G-M and N-G-M with that of G suggests that the insertion of MWCNT does not affect the diffraction peak of G. After the introduction of MWCNT into G layers, the BET specific surface area increases from 442 m^2^·g^−1^ for G to 567 m^2^·g^−1^ for G-M (Figure 2e). When the G-M is doped with nitrogen, the BET specific surface area shows no obvious change. This means that MWCNTs indeed decrease the restacking of graphene layers, harvesting higher specific surface area for the uniform dispersion of Pt nanoparticles. The pore size distributions (Figure S2) were measured by the Barrett–Joyner–Halenda (BJH) model. It is obvious that three kinds of graphene supports demonstrate hierarchical porous structures, which favor better dispersion of Pt nanoparticles and rapid diffusion path for mass transfer [41]. Identical pore size distributions notwithstanding, the total pore volume for G-M and N-G-M is 2.61 and 2.52 cm^3^·g^−1^, larger than that of G (1.97 cm^3^·g^−1^). The Raman scattering studies further confirm the degree of defects in the graphene structure. As shown in Figure 2f, the D-band located at 1332 cm^–1^ originates from the disordered structure and defects, while the G-band located at 1580 cm^–1^ is ascribed to the vibration of sp^2^-bonded carbon atoms. The ratios between the D band and G band (*I*_D_/*I*_G_) for the MWCNT, G, G-M, and N-G-M supports are estimated to be 1.10, 1.21, 1.31, and 1.39, respectively. The N-G-M support exhibits the highest *I_D_/I_G_*, which is mainly related with more defective sites affected by N doping and an increase in disordered structures from the incorporation of MWCNTs within graphene layers.

**Figure 3:**
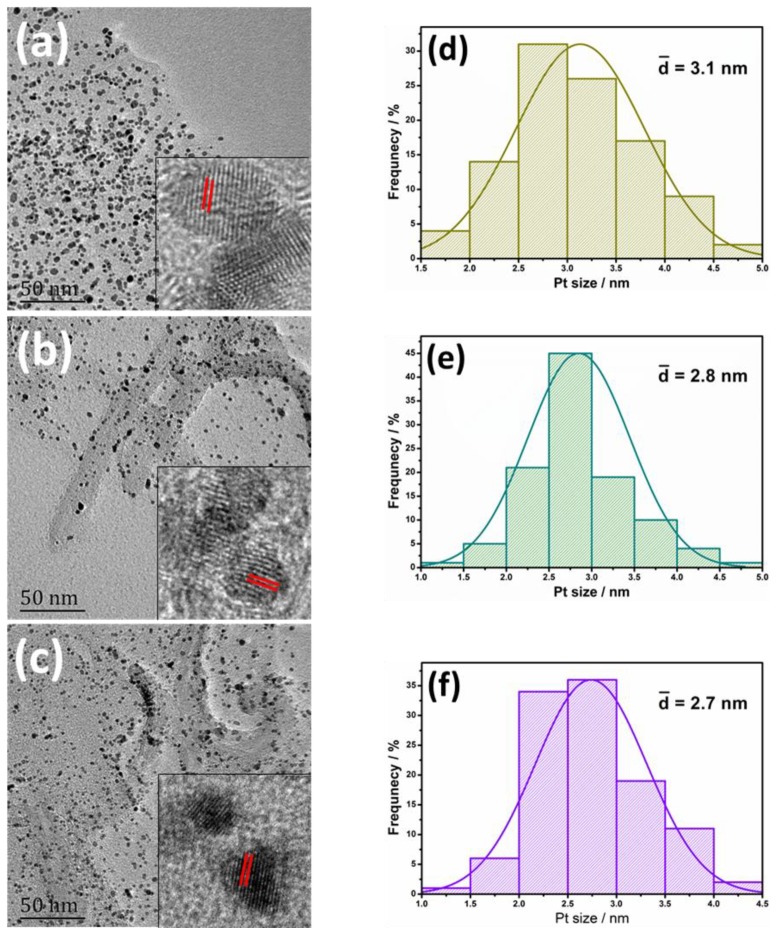
TEM images for (**a**) Pt/G, (**b**) Pt/G-M, and (**c**) Pt/N-G-M catalysts, and the corresponding particle size distribution curves for (**d**) Pt/G, (**e**) Pt/G-M, and (**f**) Pt/N-G-M catalysts. Insets show the corresponding HRTEM images.

Figure 3, Figure S3, and Figure S4 reveal the morphologies and nanostructure of Pt/M, Pt/G, Pt/G-M, and Pt/N-G-M catalysts synthesized by means of the polyol reduction method along with the JM20 catalyst. Pt nanoparticles are uniformly distributed on G-M and N-G-M hybrid supports without noticeable aggregations in the low magnification TEM images (Figure S4). High-resolution TEM analysis (Figure 3a–c insets) further demonstrates the highly crystalline features of Pt with well-resolved lattice fringes corresponding to the inter-plane spacing of 0.23 nm, which is assigned to the (111) plane of Pt. Due to the synergistic interactions between G and MWCNTs, the hybrid 3D support provides higher specific surface area for the uniform dispersion of Pt nanoparticles. As the bridge to prevent the stacking of graphene layers, Pt nanoparticles can also be deposited on the surface of MWCNTs to be the center of active sites. The histograms of the Pt nanoparticle size distributions, obtained by determining the size of about 100 randomly statistic Pt nanoparticles, are presented (Figure 3d–f). The Pt/G-M and Pt/N-G-M catalysts have a narrower and smaller distribution of Pt size than that of Pt/G, indicating the advantages of hybrid supports, which benefit the uniform and narrow deposition of Pt nanoparticles. Furthermore, Pt/N-G-M displays a smaller average size and a better dispersion of Pt nanoparticles than Pt/G-M (Figure S4). These results prove that the heteroatom N in the carbon structure of hybrid supports is beneficial for the nucleation and dispersion of Pt nanoparticles [29].

**Figure 4:**
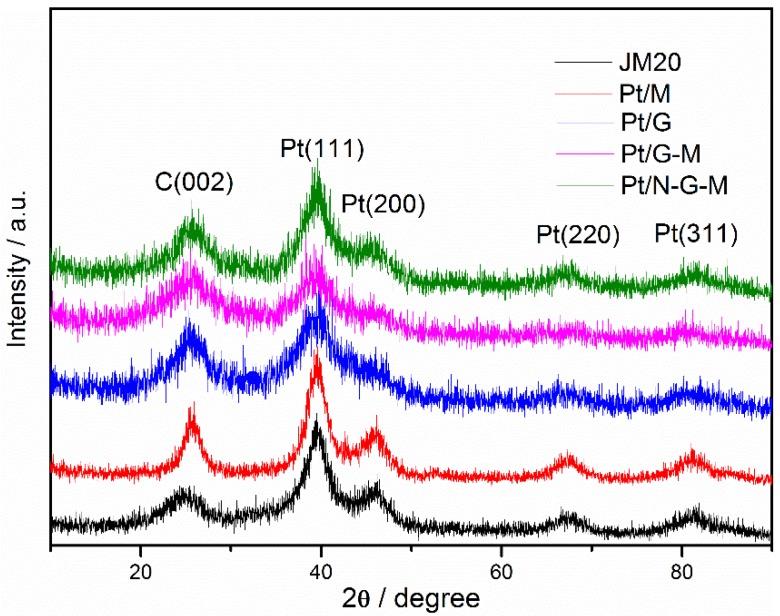
XRD patterns of JM20, Pt/M, Pt/G, Pt/G-M, and Pt/N-G-M catalysts.

To obtain the crystalline structure of as-synthesized catalysts, XRD measurements were performed and the corresponding patterns are shown in Figure 4. The peaks at approximately 39.8°, 46.4°, 67.6°, and 81.8° correspond to the (111), (200), (220) and (311) planes of Pt (JCPDS 04-0802), respectively, which are the characteristic peaks of face-centered cubic (fcc) crystalline Pt, indicating that Pt nanoparticles are in the form of a fcc-structure on different kinds of supports. 

**Figure 5:**
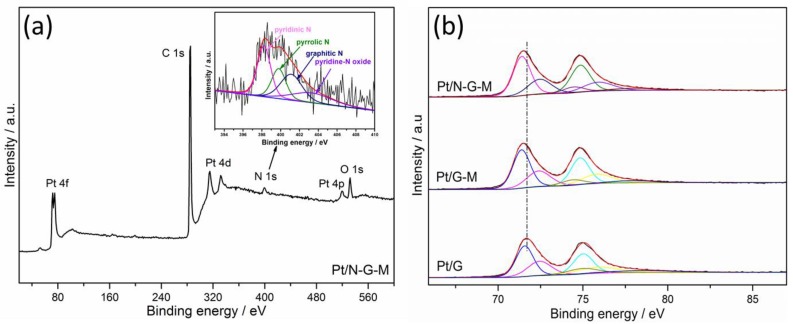
XPS spectra of Pt/N-G-M (**a**), the corresponding N 1s spectrum (inset); (**b**) the comparison of the Pt 4f binding energy of Pt/G, Pt/G-M, and Pt/N-G-M.

XPS was further conducted to reveal the surface chemical states of catalysts, with the results presented in Figure 5. The wide scan spectra of Pt/N-G-M (Figure 5a) confirm that Pt, C, and N elements are existent, and the N content was recorded to be 2.87 atom %. The high-resolution N 1s spectrum for Pt/N-G-M is shown in Figure 5a inset, which can be deconvoluted into four nitrogen functional groups: pyridinic N (398.6 eV), pyrrolic N (400.3 eV), graphitic N (401.4 eV), and pyridine-N oxide (403.3 eV) [42]. It has been well-documented that pyridinic N is capable of improving the ORR activity in the N-doped non-noble carbon materials [43,44]. In this study, it is expected that the principle pyridinic N content can improve the metal–carbon interactions and benefit the ORR activity. Pt 4f spectra for Pt/G, Pt/G-M, and Pt/N-G-M are presented in Figure 5b, which can be split into 4f_7/2_ and 4f_5/2_ states due to spin orbital splitting. The Pt^0^ at 71.4 eV (4f_7/2_) and 74.7 eV (4f_5/2_) are the predominant peaks, while the peaks at 72.4 eV and 75.8 eV as well as 75.2 eV and 78.6 eV are assigned to Pt in the 2^+^ and 4^+^ states, respectively. Table S1 shows the percentage of three Pt species calculated from the peak fitting. It is noteworthy that Pt/G-M and Pt/N-G-M have higher Pt^0^ concentrations than Pt/G, which may be beneficial for electrochemical activity [45]. Furthermore, Pt/G-M and Pt/N-G-M exhibit a negative shift of binding energy compared to Pt/G (Figure 5b), indicating the synergistic effect between G and MWCNTs, as well as the specific interactions between N-doped graphene hybrid and Pt nanoparticles because of electron donation transferred from N to Pt [46]. The negative shift for binding energy weakens the –OH adsorption species owing to the downshift of the D-band center, resulting in increased electrocatalytic performance of Pt nanoparticles towards ORR [47]. 

**Figure 6:**
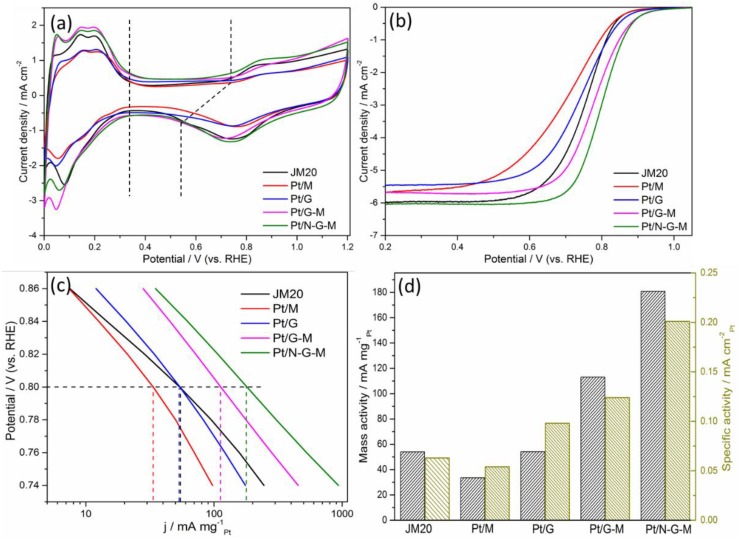
CV curves for (**a**) JM20, Pt/M, Pt/G, Pt/G-M, and Pt/N-G-M in 0.1 M HClO_4_ with a potential scan rate of 50 mV·s^−1^; (**b**) LSV curves at 5 mV·s^−1^; (**c**) MA for the ORR of JM20, Pt/M, Pt/G, Pt/G-M, and Pt/N-G-M catalysts; (**d**) MA and SA calculated at 0.80 V.

Figure 6a illustrates the typical CVs of commercial JM20, Pt/M, Pt/G, Pt/G-M, and Pt/N-G-M catalysts. It can be observed that all the catalysts demonstrate three distinct characteristic potential regions, namely a hydrogen adsorption/desorption region, a double-layer region, and a Pt oxidation/reduction region (three regions marked by the dotted lines). Notably, Pt/G-M and Pt/N-G-M show higher hydrogen adsorption/desorption than Pt/G and Pt/M, highlighting the positive effect of the insertion of MWCNTs into graphene layers. When the G nanosheets are intercalated by MWCNTs as the hybrid support for Pt, the hydrogen adsorption/desorption area of Pt/G-M and Pt/N-G-M catalysts improves owing to the boosted utilization of catalytic Pt nanoparticles. The coulombic charge for hydrogen desorption (Q_H_) in the potential range of 0–0.4 V was used to calculate the ECSA of the catalysts on the electrodes. After correcting for the double-layer charging current from the CV curves, the values of ECSA for JM20, Pt/M, Pt/G, Pt/G-M, and Pt/N-G-M are 86.6, 62.3, 55.3, 91.5, and 90.1 m^2^·g^−1^, respectively. Pt/G-M and Pt/N-G-M catalysts exhibit the highest ECSA, though G and MWCNT utilized as the sole support do not perform well in the CV. The increased ECSA for Pt/G-M and Pt/N-G-M catalysts is attributed to the intercalation of MWCNTs into graphene layers, exposing more uniform and narrowly sized Pt nanoparticles in the electrochemical reactions. Even compared to the benchmark JM20 catalyst, Pt/G-M and Pt/N-G-M are superior, suggesting the superiority of hybrid supports for depositing Pt nanoparticles compared with CB.

The ORR activity polarization curves for all the catalysts were measured by RDE in the O_2_-saturated electrolyte, and the LSV results are shown in Figure 6b. The pure G as a support for Pt nanoparticles displays inferior ORR performance, especially for the mixed kinetic-diffusion region and diffusion-limiting currents, which is attributed to the fact that the pure G-based catalysts inhibit the oxygen reduction rate, the results of which are concordant with our previous research [33] and other research [22]. This is partly originating from the aggregation of 2D planar structure of graphene and the aggregated film structure formed in the synthesis process of graphene, both of which hinder the mass transfer process [48]. Interestingly, when MWCNT is intercalated into G-based Pt catalysts, the mixed kinetic-diffusion region and diffusion-limiting currents of Pt/G-M and Pt/N-G-M catalysts are dramatically improved, indicating that the ORR activity recovers in a way. The observed improvement is ascribed to the fact that the inserted MWCNTs not only enlarge the gaps between graphene sheets but also facilitate the electron transfer, providing accessibility for the reactant to Pt nanoparticles and accelerating the oxygen reduction rate during the triple-phase reactions. The intrinsic ORR activity can be evaluated from the mass activity (MA) and specific activity (SA) calculated based on the Koutecky–Levich equation: 1/i = 1/i_k_ + 1/i_d_, where i_k_ is the kinetic current, and i_d_ is the diffusion-limiting current [49]. Figure 6c,d show the results of different catalysts. The ORR activity increases in the order of Pt/M<Pt/G<JM20<Pt/G-M<Pt/N-G-M, illustrating coincident results with the CV results. Pt/G-M and Pt/N-G-M exhibit higher onset potentials and half-wave potentials than that of Pt/G because of the intercalation of MWCNTs into graphene nanosheets. The half-wave potentials for Pt/G-M and Pt/N-G-M are 0.799 and 0.780 V, 46 and 27 mV higher than that of JM20 catalyst, respectively. With regard to the mass activity, Pt/G-M and Pt/N-G-M are 2.1 and 3.4 times higher than JM20, indicating the better performance of G-M hybrid-supported Pt catalysts. Compared with Pt/G-M, the ORR activity of Pt/N-G-M could be further improved by the doping of N into the G-M hybrid, indicating that the modified electron structure of N-G remarkably improves the Pt–graphene interactions. The electrochemical impendence spectra (Figure S5) demonstrate that the values for charge transfer resistance of JM20, Pt/G, Pt/G-M, and Pt/N-G-M are 30.3, 37.5, 25.7, and 24.2 Ω, respectively. The lower charge transfer resistance for Pt/G-M and Pt/N-G-M is beneficial for the charge transfer process, which is derived from the advantage of the 3D interconnected structure and better electrical conductivity. On the other hand, the methanol tolerance of the catalysts is of great importance for the ideal ORR catalysts applied in practical fuel cells. The ORR activities of Pt/N-G-M and JM20 are further tested in the saturated 0.1 M HClO_4_ and 0.1 M CH_3_OH electrolyte. As shown in Figure S6, the large current peak for JM20 at 0.67 V suggest the ORR current is greatly affected by the methanol [50]. In a striking contrast, Pt/N-G-M only shows a small negative shift for the half-wave potential, which proves the excellent catalytic selectivity for ORR against the methanol oxidation of Pt/N-G-M. 

**Figure 7:**
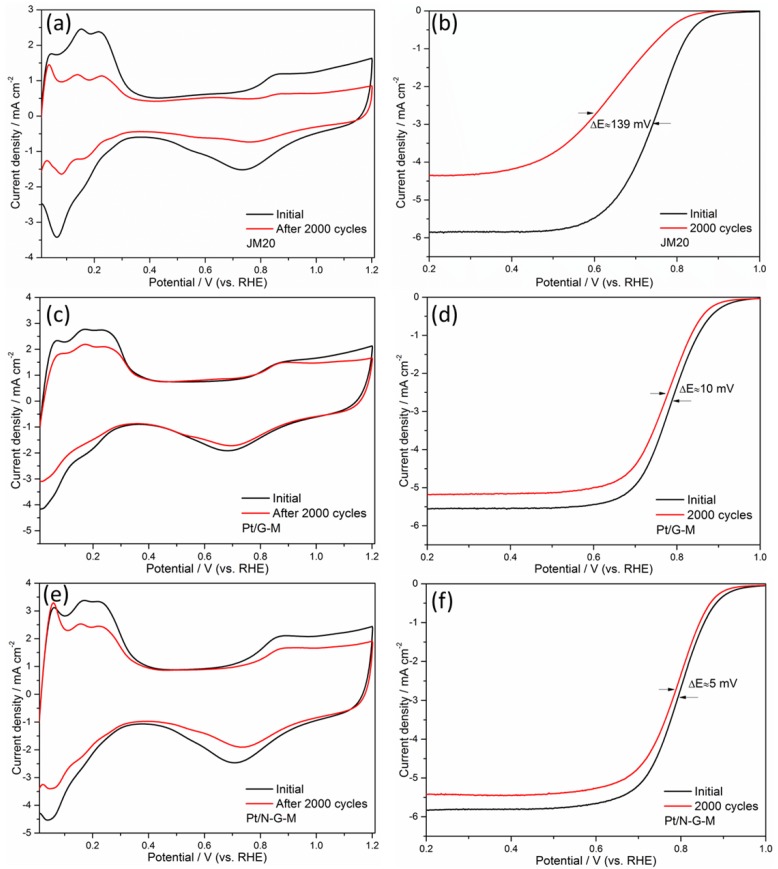
CV curves of (**a**) JM20, (**c**) Pt/G-M, and (**e**) Pt/N-G-M before and after ADT, CV recorded at a scan rate of 100 mV·s^−1^ in 0.1 M HClO_4_. LSV curves for (**b**) JM20, (**d**) Pt/G-M, and (**f**) Pt/N-G-M before and after ADT, LSV recorded at a scan rate of 5 mV·s^−1^ in 0.1 M HClO_4_.

The ADT tests were employed to evaluate the long-term stability of the as-prepared catalysts, in which the potential was cycled ranging from 0 to 1.2 V in 0.1 M N_2_-saturated electrolyte at 100 mV·s^−1^. The changes for the CV and LSV curves are shown in Figure 7. Continuous cycles lead to the deactivation of the catalysts because of the aggregation and dissolution of Pt nanoparticles, which can be gleaned from the ORR performance. When the ECSA values of catalysts are normalized to the respective initial value, Pt/G-M and Pt/N-G-M show a loss of 31.2% and 27.3%, superior to the 55.6% loss for JM20 catalyst, indicating that the prepared Pt/G-M and Pt/N-G-M manifest much better durability than JM20. In addition, LSV cycling stabilities of JM20, Pt/G-M, and Pt/N-G-M catalysts in the ORR were also examined. As displayed in Figure 7b,d,f, the JM20 catalyst has a noteworthy 139 mV negative shift for the half-wave potential after ADT, whereas only 10 and 5 mV losses are shown for Pt/G-M and Pt/N-G-M, respectively. The results are expected because the G-based catalysts can withstand the extreme electrochemical circumstance, rendering Pt nanoparticles to maintain their activity. It is noteworthy that Pt/N-G-M performs better than Pt/G-M in the ADT, further proving that the heteroatom N modifies the metal–support interactions, which effectively strengthen the binding of Pt with the hybrid support. This mitigates the dissolution, aggregation, and detachment of the Pt nanoparticles. The remarkable retention for ORR is meaningful for the long-term use of catalysts in PEMFCs.

**Figure 8:**
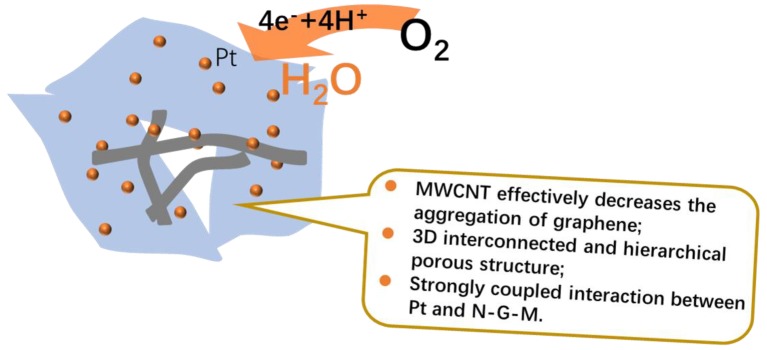
The proposed reasons for the enhanced catalytic activity of Pt/N-G-M catalysts for ORRs.

With the aim to well explain the superb electrocatalytic activity of Pt/N-G-M catalyst for ORR, several reasons are proposed according to the above results (Figure 8). Firstly, MWCNTs can be well inserted into the graphene sheets through the one-pot Hummers method, rendering graphene with higher specific surface area, which is beneficial for the deposition and exposure of Pt nanoparticles. Moreover, the hierarchical porous structure and higher specific surface area can provide fast mass transfer and electron transport kinetics, which make the reactant molecules more accessible to catalysts. Lastly, the heteroatom N can modify the electron structure of graphene, being an anchoring site for Pt nanoparticles. The strongly coupled interactions between Pt and N-G-M effectively promote the electrocatalytic activity and durability on account of the changed electron configuration. As a result of integrating these advantages above, the resultant Pt/N-G-M catalyst with unique multi-component 3D interconnected network structure possesses remarkably prominent catalytic activity, which may be a potential electrocatalyst in fuel cells.

## 4. Conclusions 

In conclusion, a novel nitrogen-doped G-M hybrid support has been uniquely designed for the deposition of uniform and narrowly sized Pt nanoparticles. The MWCNTs, as the spacer of graphene sheets, increase the specific surface area, facilitating the utilization of Pt, which is beneficial for the improvement of ORR performance. The as-fabricated Pt/G-M and Pt/N-G-M perform better than Pt/G and Pt/M, evidenced by the half-cell tests. Notably, Pt/N-G-M exhibits superior ORR activity and durability as compared to the commercial JM20 catalyst, indicating that the N-G-M support is an alternative to commercial carbon black. The highly efficient and durable Pt/N-G-M electrocatalyst paves the way for the potential application in PEMFCs.

## Supplementary Materials

The following are available online at http://www.mdpi.com/1996-1944/11/4/642/s1, Figure S1: SEM images (a,b) and TEM images (c,d) of GO-MWCNT. Figure S2: The pore size distributions of G, G-M, and N-G-M supports. Figure S3: TEM images for (a) JM20 and (c) Pt/M catalysts. The corresponding particle size distribution curves for (b) JM20 and (d) Pt/M catalysts. Figure S4: Low magnification TEM images for (a) Pt/G, (b) Pt/G-M, and (c) Pt/N-G-M catalysts. Figure S5: Nyquist plots of EIS for JM20, Pt/G, Pt/G-M, and Pt/N-G-M recorded in 0.1 M HClO4. Figure S6: ORR polarization curves for JM20 and Pt/N-G-M catalysts in 0.1 M HClO_4_ + 0.1 M CH_3_OH with a potential scan rate of 5 mV·s^−1^. Table S1: Results of the fits of Pt 4f spectra, values given in percentage of total intensity.
