# Supplementary material for: Synergistic Effect of Nitrogen Doping and MWCNT Intercalation for the Graphene Hybrid Support for Pt Nanoparticles with Exemplary Oxygen Reduction Reaction Performance
Source: Materials (Basel). 2018 Apr 22;11(4):642. doi: 10.3390/ma11040642 (PMC5951526; doi:10.3390/ma11040642)
Supplement: Supplementary file 1 [file materials-11-00642-s001.pdf]

Supplementary Material

Synergistic Effect of Nitrogen Doping and MWCNT Intercalation for the Graphene Hybrid Support for Pt Nanoparticles with Exemplary Oxygen Reduction Reaction Performance

Kang Fu, Yang Wang, Ying Qian, Linchang Mao, Junhong Jin, Shenglin Yang and Guang Li *

State Key Laboratory for Modification of Chemical Fibers and Polymer Materials, College of Materials Science and Engineering, Donghua University, Shanghai, China; fuk_1992@126.com (K.F.); DHUWangY@163.com (Y.W.); viskyying@163.com (Y.Q.); 2140278@mail.dhu.edu.cn (L.M.); jhkin@dhu.edu.cn (J.J.); slyang@dhu.edu.cn (S.Y.)

* Correspondences: lig@dhu.edu.cn; Tel.: +86-21-6779-2830; Fax: +86-21-6779-2855

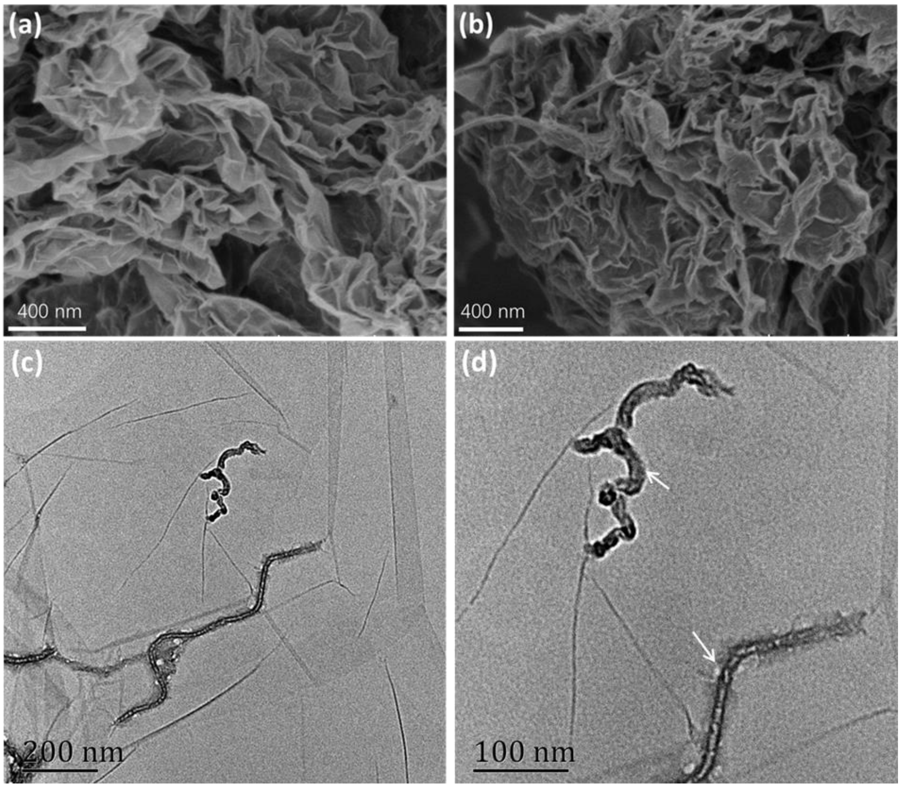

Figure S1. SEM images (**a**,**b**) and TEM images (**c**,**d**) of GO-MWCNT.

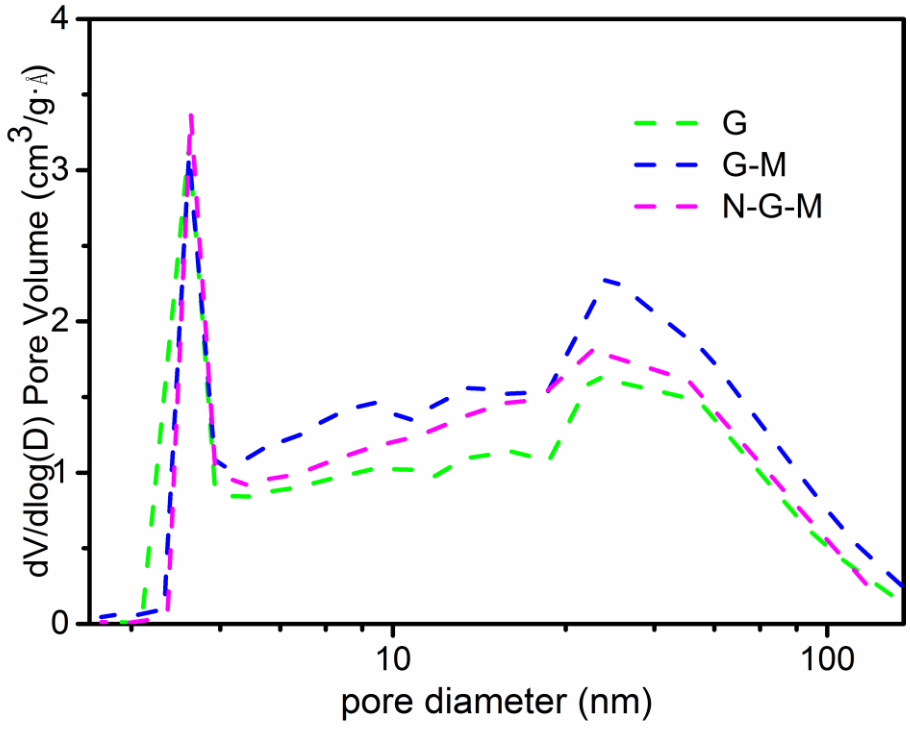

Figure S2. The pore size distributions of G, G-M, and N-G-M supports.

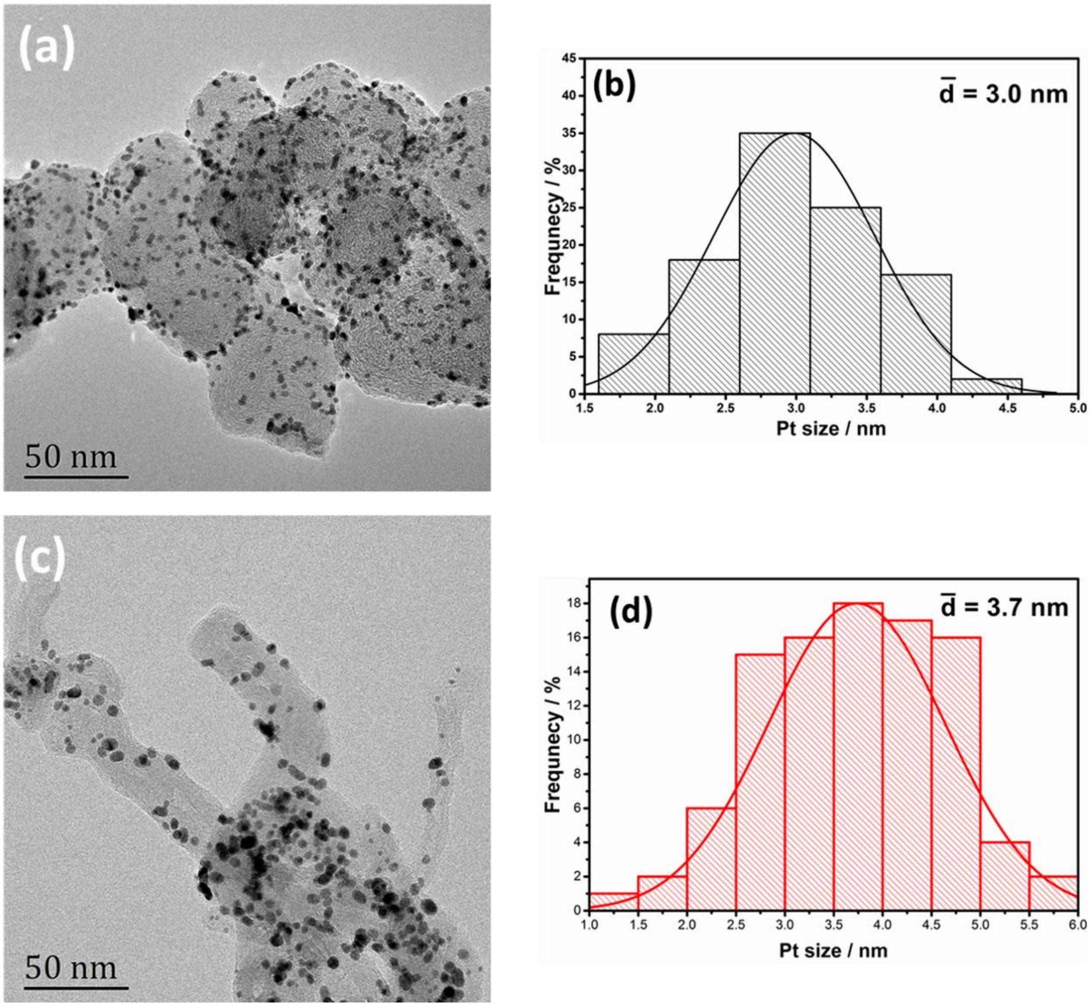

Figure S3. TEM images for (**a**) JM20 and (**c**) Pt/M catalysts. The corresponding particle size distribution curves for (**b**) JM20 and (**d**) Pt/M catalysts.

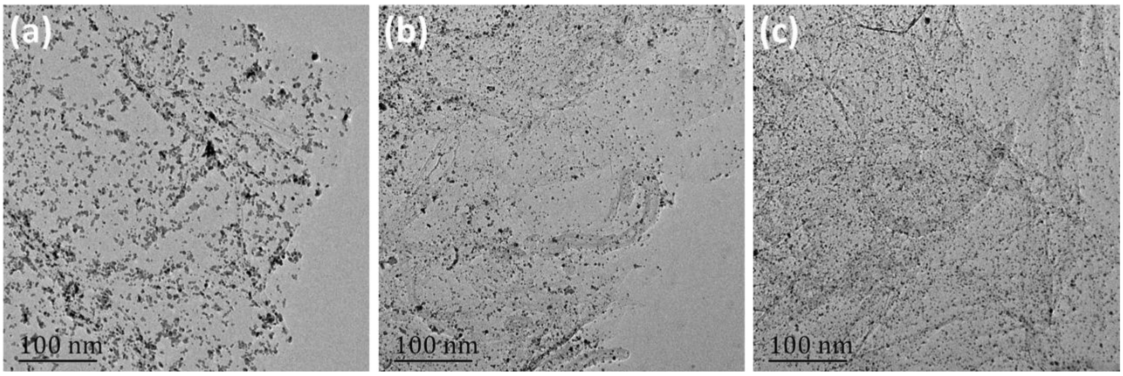

Figure S4. Low magnification TEM images for (**a**) Pt/G, (**b**) Pt/G-M, and (**c**) Pt/N-G-M catalysts.

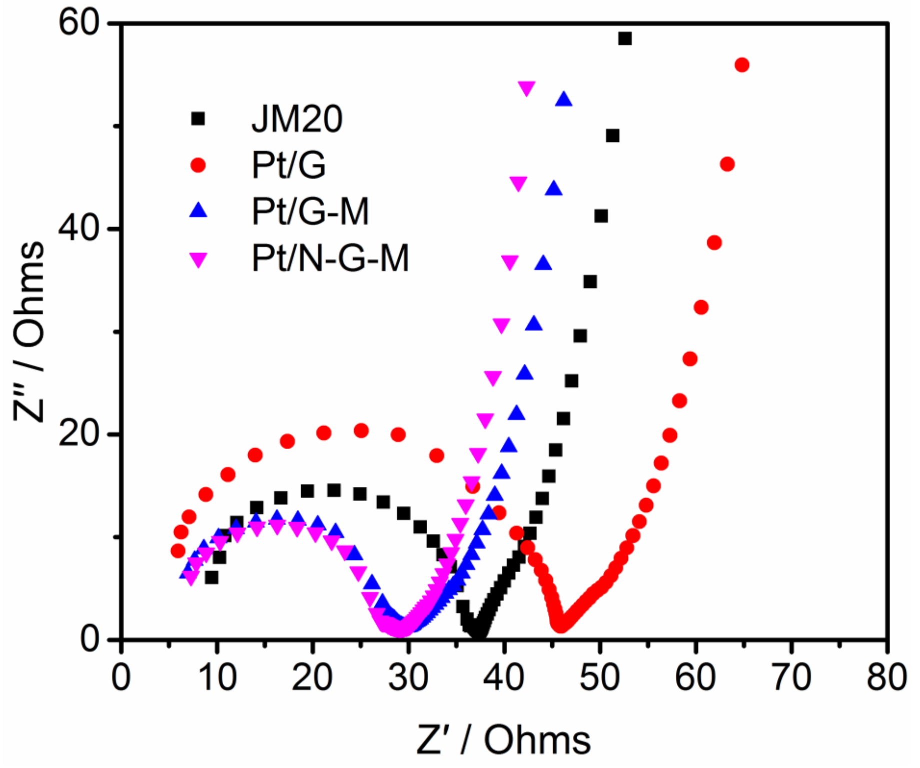

Figure S5. Nyquist plots of EIS for JM20, Pt/G, Pt/G-M and Pt/N-G-M recorded in 0.1 M $HClO_4$.

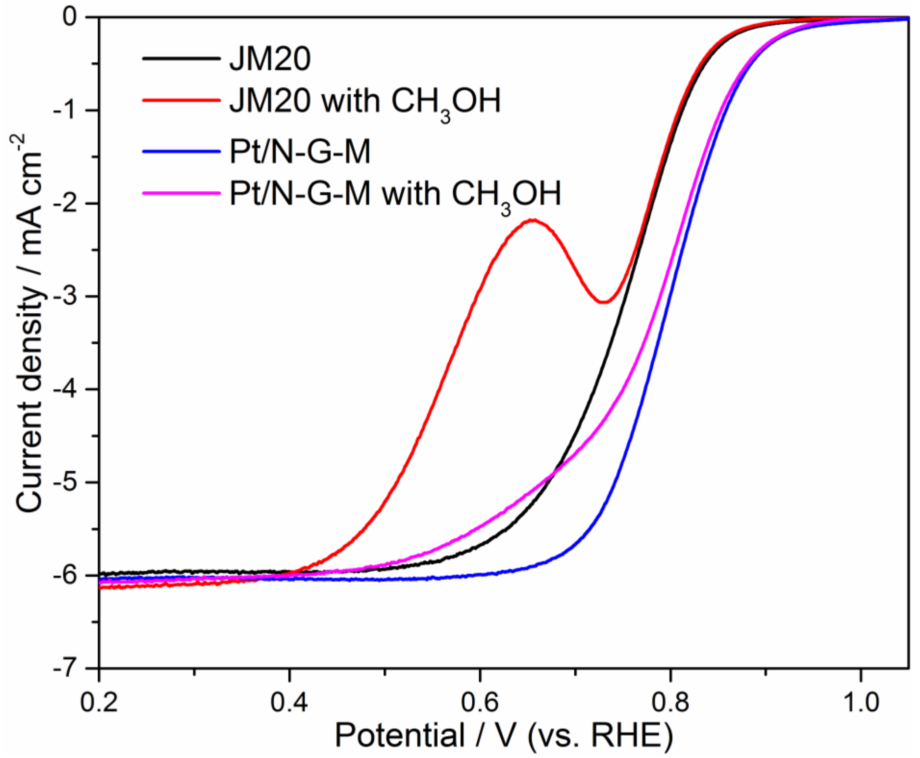

Figure S6. ORR polarization curves for JM20 and Pt/N-G-M catalysts in 0.1 M $HClO_4$ + 0.1 M CH_3OH with a potential scan rate of 5 $mV{\cdot}s^{-1}$.

Table S1. Results of the fits of Pt 4f spectra, values given in percentage of total intensity.

Catalyst	Pt Species		
	Pt^0	Pt^{2+}	Pt^{4+}
Pt/G	51.1	32.6	16.3
Pt/G-M	57.2	32.1	10.7
Pt/N-G-M	59.6	30.2	10.2
